# Exploring a Third Confirmed Case of Hemoperitoneum following Open Inguinal Hernia Repair Caused by Sampson Artery Hemorrhage

**DOI:** 10.1155/2017/1487526

**Published:** 2017-04-10

**Authors:** Jordan Hebert, Manoj Jagtiani, Alexander Hsu, David Schmelzer, Fred Wolodiger

**Affiliations:** ^1^Hackensack University Medical Center, Hackensack, NJ 07601, USA; ^2^Department of Surgery, Englewood Hospital, Englewood, NJ 07631, USA; ^3^Touro College of Osteopathic Medicine, New York, NY 10027, USA

## Abstract

Hemoperitoneum is a rare complication of open inguinal hernia repair. This is the third reported case of this complication attributed to the same bleeding source: Sampson's artery. Sampson's artery courses along the round ligament of the uterus in the inguinal canal of females, originating from the arcade formed between the uterine and ovarian arteries. Usually obliterated in postembryonic development, this artery can persist in some adult female patients. Disruption of Sampson's artery can lead to hemoperitoneum following ligation of the uterine round ligament during open inguinal hernia repair in females. This case report describes a third confirmed case of hemoperitoneum complicating an open inguinal hernia repair. We review all three reported cases to date and discuss the recurring signs, symptoms, epidemiologic factors, and diagnostic findings associated. Our review suggests that females of childbearing age, particularly those in the peripartum period, are most at risk of developing this rare complication.

## 1. Introduction

Bleeding complications following inguinal hernia repairs are rare. The European Hernia society recommends that women should be operated by laparoscopic technique depending on surgical expertise. However, the Lichtenstein technique, once the recommended technique [1], is still commonly used and was utilized in this case due to its low recurrence and complication rate [2]. Hematomas are reported to occur in 1.3% of inguinal repair cases using the Lichtenstein technique and are the most common bleeding complication [3]. In this case, we present a patient who had an open inguinal hernia mesh repair using the Lichtenstein technique, complicated by postoperative intraperitoneal hemorrhage requiring reoperation. A literature review reveals only two confirmed previous cases [4, 5]. While a third case has alluded to this particular complication, it has been disregarded by previous scholarly journals as “confirmed [6].” Bleeding, similar to the previous cases [4, 5], was found to originate from the artery of Sampson, a vessel which is normally obliterated in postembryonic development but which can persist as a branch of the uterine artery that runs along the length of the uterine round ligament in adult females.

## 2. Case Report

Our patient is a 38-year-old female with past medical history significant for hypothyroidism and a cesarean section six months ago, who had same-day surgery for elective repair of a symptomatic right inguinal hernia. The patient had episodic pain in the right inguinal region and physical examination confirmed the presence of a right inguinal hernia. An open right inguinal hernia repair was performed using the Lichtenstein technique. During the procedure, diffuse weakness of the inguinal canal floor was encountered. The round ligament was ligated with 2-0 vicryl suture and excised. Polypropylene mesh was secured in with 2-0 prolene suture. Intraoperative bleeding was minimal with excellent hemostasis noted. The patient was discharged following an unremarkable postgeneral anesthesia recovery.

At midnight on the day of the procedure, the patient presented to the emergency department with severe right upper quadrant pain radiating to the right shoulder, worsened by both the supine position and deep inspiration, which began six hours after the operation. On physical examination, the patient's abdomen was noted to be mildly distended with no peritoneal signs. There was no hematoma involving the hernia wound. The patient was found to be anemic, with increasing tachycardia, normal blood pressure, and hemoglobin falling from 14.2 g/dL preoperatively to 9.7 g/dL on emergency department presentation. Furthermore, a hemoglobin level of 8.8 g/dL was found on repeat examination. An abdominal CT scan was obtained which revealed significant hemoperitoneum (see the appendix).

Emergency diagnostic laparoscopy was performed, revealing a large amount of coagulated and liquid blood that was estimated to total 1 L after evacuation. The bleeding was noted to originate from the internal portion of the inguinal canal, which was grasped and cauterized, completely stopping the active bleeding. The patient was noted to be hemodynamically stable during the course of the operation. The patient made a rapid recovery, with no further bleeding.

## 3. Discussion

Postoperative hemoperitoneum after open inguinal hernia repair is a rare occurrence. After an extensive literature review conducted through PubMed, EBSCO, and Proquest using the terms “inguinal hernia,” “hemoperitoneum,” “complication,” “bleeding,” and “hemorrhage,” we found only two confirmed credible cases documenting this complication. These reports were published in 2006 and 2016. The rarity of this complication is well documented in the hernia literature. Postoperative complications include “hematoma or seroma development (2–13.6%), urinary retention or infection (0–2.6%), wound infection (0–1.8%), and orchitis (1–1.6%) [2, 4, 7–9].” Intraoperative vessel injury is within a range of .002–.8% [4]. Furthermore, vasculature injury commonly includes “the cremasteric artery, internal spermatic artery, branches of the inferior epigastric vessels, deep circumflex artery, and external iliac vessels [4].” The etiology of postinguinial repair hemorrhage is rarely the Sampson artery. This was the etiology in our patient.

This case report, as well as the publications of 2006 and 2016, yield three documented cases of hemoperitoneum following open inguinal repair. It is significant to note that several commonalities have been noted for a possible role in future diagnosis. These cases shared major noteworthy characteristics:

1. They were all traced to a bleeding segment of the artery of Sampson from an avulsed portion of the uterine round ligament.
2. Patients were all females of childbearing age, with 2 of the 3 patients recently pregnant.
3. Patients presented with abdominal distension and pain within 24 hours of postoperative discharge.

The above commonalities may serve as loose distinguishing characteristics shared by cases developing hemoperitoneum subsequent to open inguinal hernia repair, as not enough literature supports a definitive set of shared characteristics. It is interestingly significant to note that that the patients were all female of childbearing age. Two of the three patients had, in fact, recently had pregnancies which may suggest that pregnancy-related vessel proliferation and hypertrophy may play a role in this specific complication; furthermore, it may suggest that hormones serve as the etiology in the development of the issue. One may establish CT and diagnostic laparoscopy as optimal diagnostic modalities with diagnostic laparoscopy serving as a prime treatment with minimally ensuing complications. The above cases establish that a major postoperative hemorrhage may ensue when ligature of Sampson's artery is not properly performed. It is of significance to note that the prevalence of this complication and possibly a mode of prevention, using the currently recommended laparoscopic repair approach, may be lower than that of the Lichtenstein approach as laparoscopic repair would provide superior visibility of a transected vessel and round ligament transection is often less necessary in a laparoscopic approach.

## 4. Conclusion

Inguinal hernia repair remains one of the most common surgical procedures performed, often with minimal complications. With this case being a confirmed third reported instance of hemoperitoneum resulting from inguinal hernia repair, it is a rare but noteworthy complication that requires awareness for timely diagnosis and treatment in future cases.

## Figures and Tables

**Figure 1:**
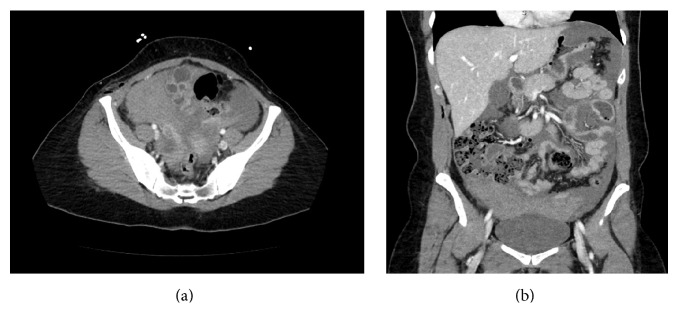
Axial (a) and coronal (b) abdominal CT scan showing hemoperitoneum.

## Appendix

See Figure 1.
